# Effect of Europium Doping on the Structural, Morphological, and Luminescent Properties of Y_3_Al_5_O_12_ Phosphors Produced by Combustion Method

**DOI:** 10.1007/s10895-025-04319-6

**Published:** 2025-04-24

**Authors:** Fikadu Takele Geldasa, Habtamu Fekadu Etefa, Francis Birhanu Dejene

**Affiliations:** Department of Chemical and Physical Sciences, Walter Sisulu University, Private Bag X1, Mthatha, 5117 South Africa

**Keywords:** Y_3_Al_5_O_12_, Photoluminescence, Luminescence, Red-orange Emission, Eu^3+^ Concentration

## Abstract

In this study, red-emitting Y_3_Al_5_O_12_:Eu^3+^ phosphor was successfully synthesized using a solution-combustion method. The structural characterization through XRD confirmed the formation of a well-crystallized Y_3_Al_5_O_12_ structure. SEM images revealed the formation of an agglomerate structure, while FTIR spectra identified the chemical bonds present in the phosphor. EDS further confirmed the presence of the expected elements in the composition. PL spectra exhibited characteristic emission peaks at 592 nm, 615 nm, and 628 nm, corresponding to transitions from the ^5^D_0_→^7^F_1_, ^5^D_0_→^7^F_2_ and ^5^D_0_→^7^F_3_, respectively, with the maximum emission observed at 615 nm. The luminescence intensity was found to increase with increasing Eu^3+^ doping concentrations up to 0.8%, after which a concentration quenching effect occurred, leading to a decrease in emission intensity at higher doping levels (1.2%). These findings demonstrate the potential of Y_3_Al_5_O_12_:Eu^3+^ as an efficient red-emitting phosphor and underscore the importance of optimizing doping concentrations for enhanced luminescent performance.

## Introduction

Yttrium aluminum garnet (Y_3_Al_5_O_12_, YAG) is a widely studied inorganic compound known for its excellent physical and optical properties, making it suitable for a broad range of applications, from traditional structural ceramics to advanced photonic devices [1–4]. Rare earth doped YAG has potential applications in the field of luminescence due to its excellent mechanical strength, high thermal conductivity, high radiation resistance and high temperature resistance [5, 6]. YAG possesses garnet type crystal structure and it is excellent host materials for rare earth ions doped lasers [1, 7, 8]. It has also widely used in scintillation, cathode-ray tubes (CRT), plasma display panels (PDPs), field emission displays (FEDs), vacuum fluorescent displays (VFDs), and electroluminescent devices [7, 9]. The versatility of YAG as a host material is further enhanced by its ability to be doped with various rare-earth elements, including europium (Eu), terbium (Tb), cerium (Ce), dysprosium (Dy), and thulium (Tm), to produce full-color phosphors with tailored optical properties [8, 10–13]. Among these doped materials, YAG: Eu^3+^ phosphors, which emit bright red light, have attracted significant attention for their potential applications in display technologies and lighting [14, 15].

For YAG: Eu^3+^ phosphor synthesis, solid state reaction is the commonly used method. However, this method has some disadvantages such as high temperature requirement (~ 1600 ^0^C) and it is huge challenge to control phase purity, uniform morphology and size in the produced samples [6, 16]. Currently, the solution chemistry methods such as sol-gel, precursor decomposition, co-precipitation and solution combustion method have investigated to prepare phosphor materials due to their simple procedures, low temperature, low cost, uniform sample morphology and easy to phase control. Among the various chemical methods, the solution combustion method stands out as a promising technique for synthesizing phosphor powders due to its simplicity, energy efficiency, and ability to achieve high-quality products at relatively low temperatures [17]. This method involves the exothermic reaction between metal nitrates and a fuel source in solution, leading to rapid combustion and the formation of highly homogeneous and pure powder materials [18]. The low synthesis temperature and energy-saving nature of this process make it particularly attractive for large-scale industrial applications.

This study presents a novel approach to the synthesis of Y_3_Al_5_O_12_:Eu^3+^ phosphors using the solution-combustion technique, focusing on the optimization of Eu^3+^ doping concentration to enhance the structural, morphological, and luminescent properties of the material. Although previous studies have explored the synthesis of Y_3_Al_5_O_12_:Eu^3+^ phosphors through the solution-combustion method, the majority of these studies have employed high-temperature, long-duration processes, which limit the scalability and efficiency of the method. For instance, Shikao and Jiye have reported the synthesis of Eu-activated Y_3_Al_5_O_12_ phosphor using solution combustion at elevated temperatures (1200 °C) for extended durations (2 h) [19]. However, the discussions in their work were relatively basic, and there was no thorough investigation into the effects of doping concentration or the optimization of synthesis conditions. The key novelty of our present work lies in the use of a low-temperature and rapid synthesis process for the production of Y_3_Al_5_O_12_:Eu^3+^ phosphors, which has not been previously reported in detail. By significantly reducing both the synthesis temperature and reaction time, this study offers a greener and more energy-efficient approach to producing high-quality phosphors. The use of lower temperatures not only reduces energy consumption but also minimizes the risk of material degradation, providing a more sustainable alternative to traditional high-temperature methods.

Additionally, another unique aspect of this study is the systematic optimization of europium doping concentration. Unlike previous reports, this research thoroughly investigates how different levels of Eu^3+^ doping affect the structural, morphological, and luminescent properties of Y_3_Al_5_O_12_ phosphors. By exploring a range of Eu^3+^ concentrations, this study identifies the optimal doping level that maximizes the material’s luminescent efficiency while maintaining structural integrity. This detailed examination of doping effects is essential for tailoring phosphors for specific applications, particularly in the fields of lighting and display technologies. Additionally, this study employs a comprehensive characterization approach, utilizing techniques such as X-ray diffraction (XRD), scanning electron microscopy (SEM), Fourier transform infrared spectroscopy (FTIR), and photoluminescence (PL) spectroscopy.

## Experimental Details

### Materials

Yttrium nitrate hexahydrate (Y(NO_3_)_3_.6H_2_O, 99.9%, Sigma-Aldrich), aluminum nitrate nonahydrate (Al(NO_3_)_3_.9H_2_O, 99.9%, Sigma-Aldrich), europium nitrate hexahydrate (Eu(NO_3_)_3_.6H_2_O, 99.9%, Alfa Aesar), and urea (CO(NH_2_)_2_, 99.5%, Sigma-Aldrich) were used as precursor materials.

### Synthesis Procedures

The Eu^3+^ doped Y_3_Al_5_O_12_ phosphor was synthesized using the solution combustion method. First, 0.5 M of Y(NO_3_)_3_·6H_2_O, Al(NO_3_)_3_·9H_2_O, and CO(NH_2_)_2_ were dissolved in 50 mL of deionized water and stirred for 30 min. After stirring, the transparent solution was transferred to a preheated muffle furnace set at 500 °C. As the solution heated, it began to boil, undergo dehydration, and decompose, releasing large amounts of gases, including carbon oxides, nitrogen oxides, and ammonia. The substantial release of gases during the reaction disperses heat, inhibiting sintering and creating favorable conditions for the formation of a crystalline phase. This process resulted in the formation of a white, foamy, and voluminous ash, and the entire reaction took less than 5 min to complete. After cooling to room temperature, the foamy powder was crushed into a fine powder using a pestle and mortar. The resulting white powder was then used for further characterization. The rapid and vigorous reaction prevents any further growth of the particles, leading to the formation of materials without additional particle enlargement. A similar procedure was followed for doping the phosphors with Eu^3+^ ions at concentrations of 0.4, 0.8, and 1.2 mol%, by adding Eu(NO_3_)_3_·6H_2_O to the 50 mL solution.

### Characterizations

The characterization techniques employed in this study include X-ray diffraction (XRD, D8 Advanced AXS GmbH) to determine the crystalline properties of the particles. Scanning electron microscopy (SEM, Shimadzu Superscan SSX-550) was used to examine the morphology of the synthesized material. Photoluminescence measurements were conducted using a Cary Eclipse Photoluminescence Spectrophotometer, which is equipped with a 150 W xenon lamp, to obtain the excitation and emission spectra. Energy dispersive spectroscopy (EDS) was utilized to analyze the elemental composition, and Fourier Transform Infrared spectroscopy (FTIR, Bruker TENSOR 27 Series) was employed to identify the types of chemical bonds present.

## Results and Discussion

### X-ray Diffraction Analysis

**Fig. 1:**
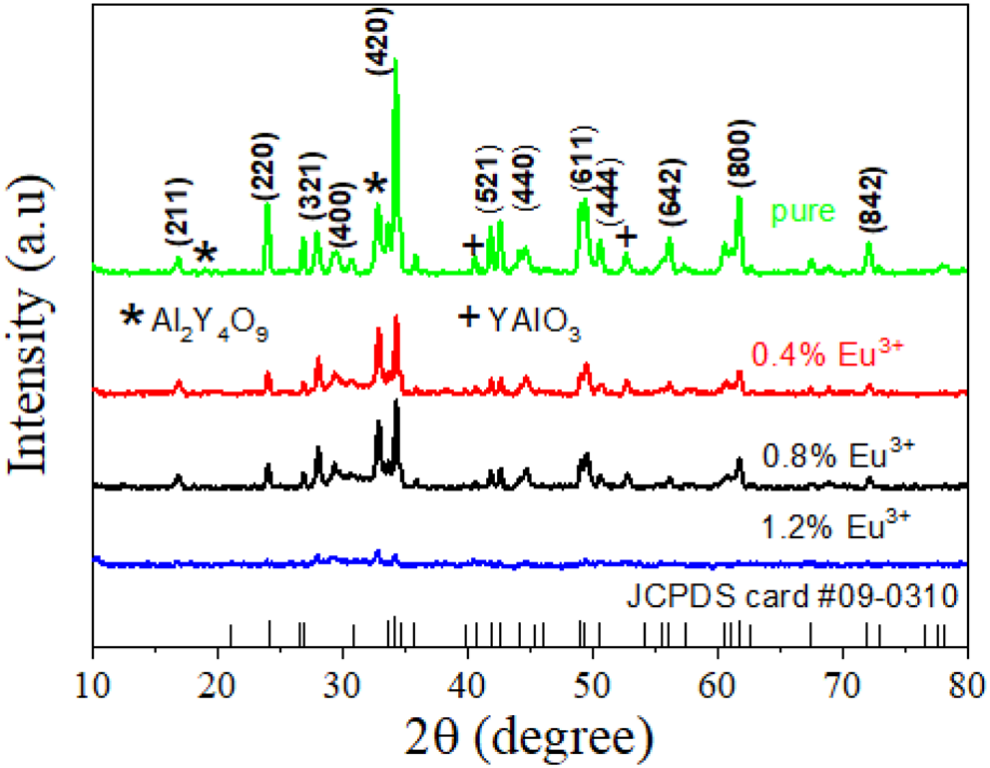
XRD patterns of pure and different concentrations of Eu^3+^ doped Y_3_Al_5_O_12_ phosphors

Figure 1 shows the X-ray spectra of different Eu^3+^ concentrations doped Y_3_Al_5_O_12_ phosphors. The diffraction peaks are in a good agreement with the cubic garnet with the standard JCPDS 09-0310. Some of the emerged diffraction peaks are indexed as (211), (220), (321), (400), (420), (521), (440), (611), (444), (642), (800) and (842). Other emerged diffraction peaks are due to some impurities which are detected during the combustion synthesis method and are attributed to other phases such as Al_2_Y_4_O_9_ and YAlO_3_. Among the indexed peaks, the (420) is a predominance peak indicating that the direction of phase orientation. When the concentrations of Eu^3+^ increases the intensity of the predominance peak of (420) indexed is decreasing which indicates that the crystallinity of Y_3_Al_5_O_12_ phosphors is decreased. Specifically, for the phosphor doped with the 1.2% of Eu, only very small intensity diffraction peaks along the (420) are appeared. Thus, except along this orientation no diffraction peaks are appeared which indicates that increasing the amount of dopant concentration led the Y_3_Al_5_O_12_:Eu^3+^ phosphor as amorphous phosphor materials.

In doping, the Eu^3+^ ions may substitute for Y^3+^ ions in the host in crystal lattice. At higher concentrations, Eu^3+^ dopant ions can cause local distortions or irregularities in the lattice, disrupting the orderly arrangement required for crystalline growth [20]. This disruption can hinder the formation of a well-ordered crystalline structure, leading to the formation of an amorphous phase instead. On the other hand, in combustion synthesis, when the Eu^3+^ dopant ions concentration increases, the exothermic reaction may become more intense, leading to rapid cooling and quenching. This rapid cooling can freeze the structure in an amorphous state before the crystals have a chance to properly form.

**Fig. 2:**
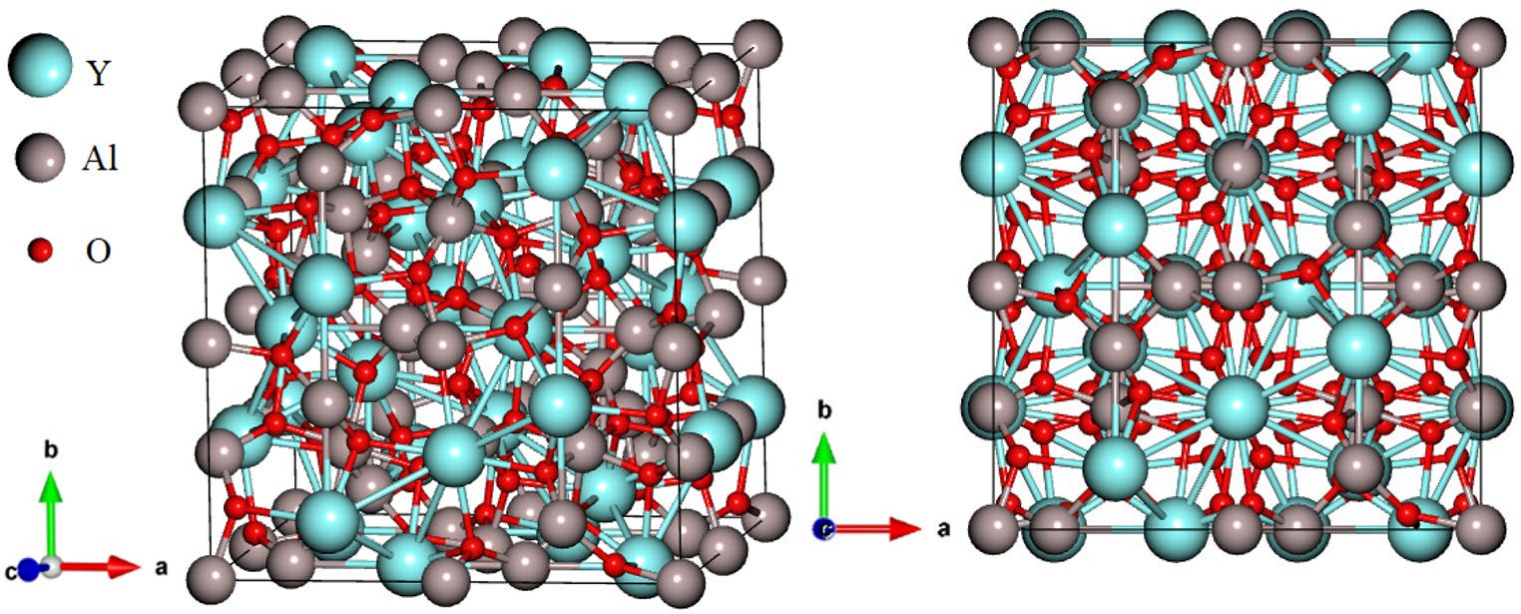
Crystal structures of Y_3_Al_5_O_12_ phosphor material in different directions

The crystal structure of Y_3_Al_5_O_12_ phosphor consisting 160 atoms is shown in Fig. 2. Y_3_Al_5_O_12_ crystallizes in the cubic *Ia̅3d* space group, characterized by a well-ordered structure where the Y^3+^ and Al^3+^ ions are arranged in distinct coordination environments with O^2−^ ions [3]. In this structure, the Y^3+^ ions are coordinated in a distorted body-centered cubic geometry, surrounded by eight equivalent O^2−^ atoms. The Y-O bond lengths are unequal, with four shorter bonds measuring 2.31 Å and four longer bonds at 2.44 Å, reflecting the distortion in the coordination geometry.

The Al^3+^ ions occupy two distinct sites within the crystal lattice. In the first Al^3+^ site, each Al^3+^ ion is bonded to four equivalent O^2−^ atoms, forming corner-sharing AlO_4_ tetrahedra. The angle between the corner-sharing octahedra is 49^0^, and all Al-O bond lengths in this site are 1.77 Å. The second Al^3+^ site is coordinated to six equivalent O^2−^ atoms, forming corner-sharing AlO_6_ octahedra, with Al-O bond lengths of 1.92 Å. The O^2−^ ions are tetrahedrally coordinated in a 4-fold geometry, where each O^2−^ ion is bonded to two equivalent Y^3+^ and two Al^3+^ atoms. This arrangement creates a highly symmetrical and stable crystal structure that contributes to the material’s overall physical and optical properties.

### SEM Analysis

**Fig. 3:**
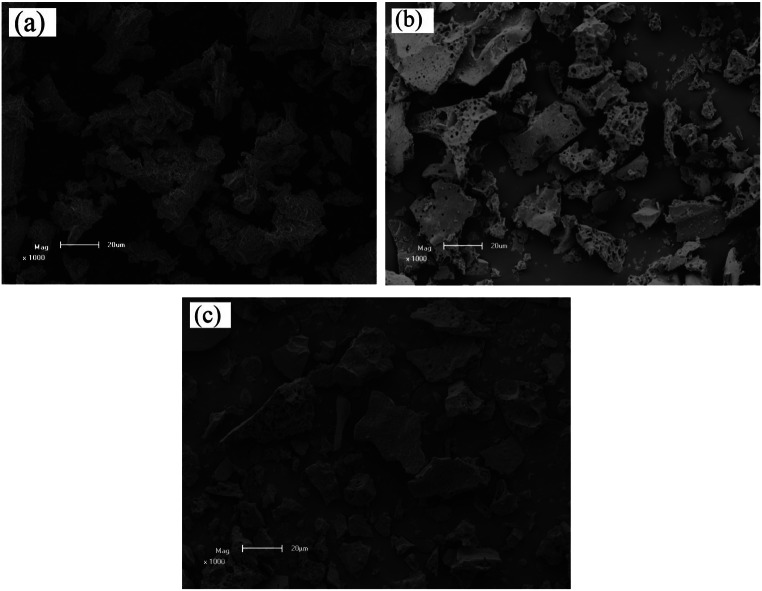
SEM photographs of (**a**) Y_3_Al_5_O_12_, (**b**) 0.4 mol% Eu doped and (**c**) 1.2 mol% Eu doped Y_3_Al_5_O_12_ phosphors

The scanning electron micrographs of pure Y_3_Al_5_O_12_ and Eu^3+^ doped Y_3_Al_5_O_12_ phosphors, with doping concentrations of 0.4 and 1.2 mol%, are presented in Fig. 3. The SEM images were captured at a magnification of 1000$\:\times\:$, providing a detailed view of the surface morphology of the phosphor particles. From the micrographs, it is evident that the phosphor exhibits an agglomerate structure, meaning that the individual particles tend to cluster together, forming larger aggregates. This is a common feature observed in the synthesis of phosphor materials using the solution-combustion method, where the rapid formation of solid particles can lead to particle aggregation [21].

In addition to the agglomeration, the SEM images reveal the presence of several small particles located around the pores. The pores themselves are a result of the high-pressure gas formation during the combustion process. As the gas escapes during the rapid combustion reaction, it leaves behind voids or pores within the material. These pores are observed to be surrounded by small particles, which likely form due to the condensation of vaporized material or the migration of particles towards regions of lower pressure. This phenomenon is consistent with the findings in the literature, where similar effects have been observed in solution-combustion-synthesized phosphors [22]. The high pressure generated during the combustion reaction is a key factor in the creation of these pores and small particles, influencing the overall morphology of the synthesized material. These morphological features, including the agglomeration and porosity, are crucial for understanding the physical properties of the phosphor material, as they can influence the material’s optical properties and performance in various applications, such as in luminescence and light-emitting devices.

### FTIR Analysis

**Fig. 4:**
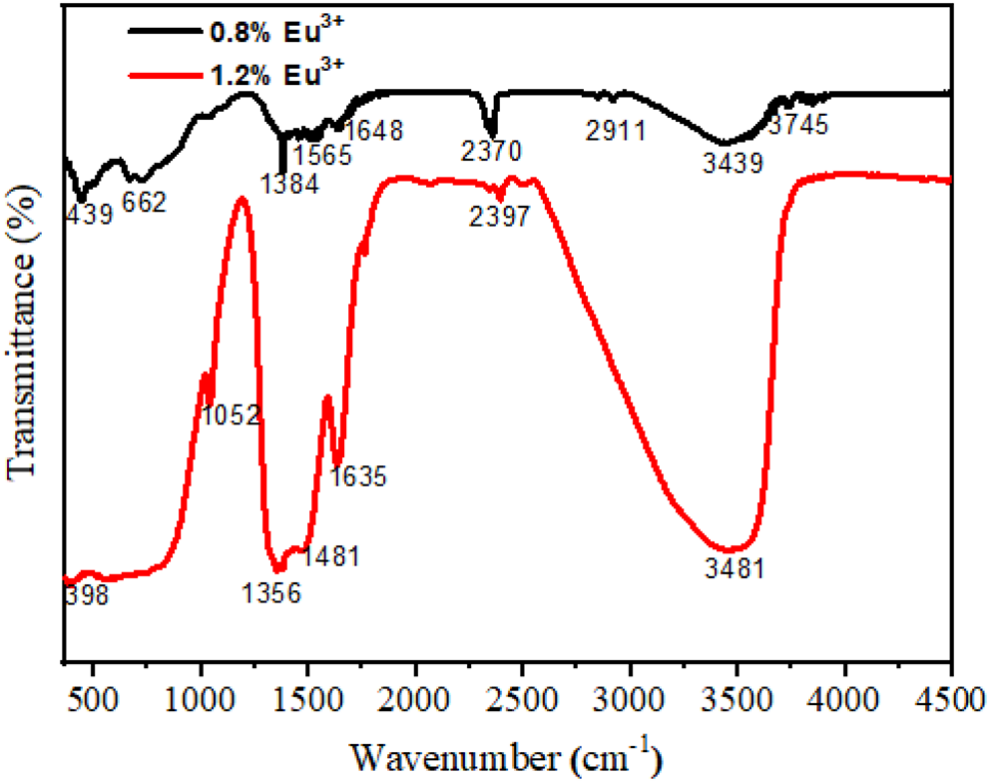
FTIR spectra of Y_3_Al_5_O_12_:Eu^3+^ phosphors

The Fourier Transform Infrared (FTIR) spectra of the Y_3_Al_5_O_12_:Eu^3+^ phosphors are shown in Fig. 4. FTIR analysis provides valuable information about the materials functional groups and bonding environment, helping to identify specific vibrations related to the phosphors structure [23]. Several common bands appear in both spectra, including the O-H band at 3439 cm^− 1^ and 3481 cm^− 1^, the H_2_O vibration bands at 1648 cm^− 1^ and 1635 cm^− 1^, and the bands at 1384 cm^− 1^ and 1356 cm^− 1^, which are attributed to NO^3−^ groups. Bands at 2370 cm^− 1^ and 2397 cm^− 1^ are observed, resulting from trace amounts of CO_2_ trapped in the combustion products. Peaks in the 300 cm^− 1^ to 1000 cm^− 1^ range are associated with metal-oxygen stretching and bending vibrations. The band at 662 cm^− 1^, seen in the 0.8% concentration is characteristic of Al-O vibrations, while the bands at 439 cm^− 1^ and 398 cm^− 1^, present in both concentrations, correspond to Y-O vibrations. These metal-oxygen absorption bands suggest the formation of the YAG phase [22].

**Fig. 5:**
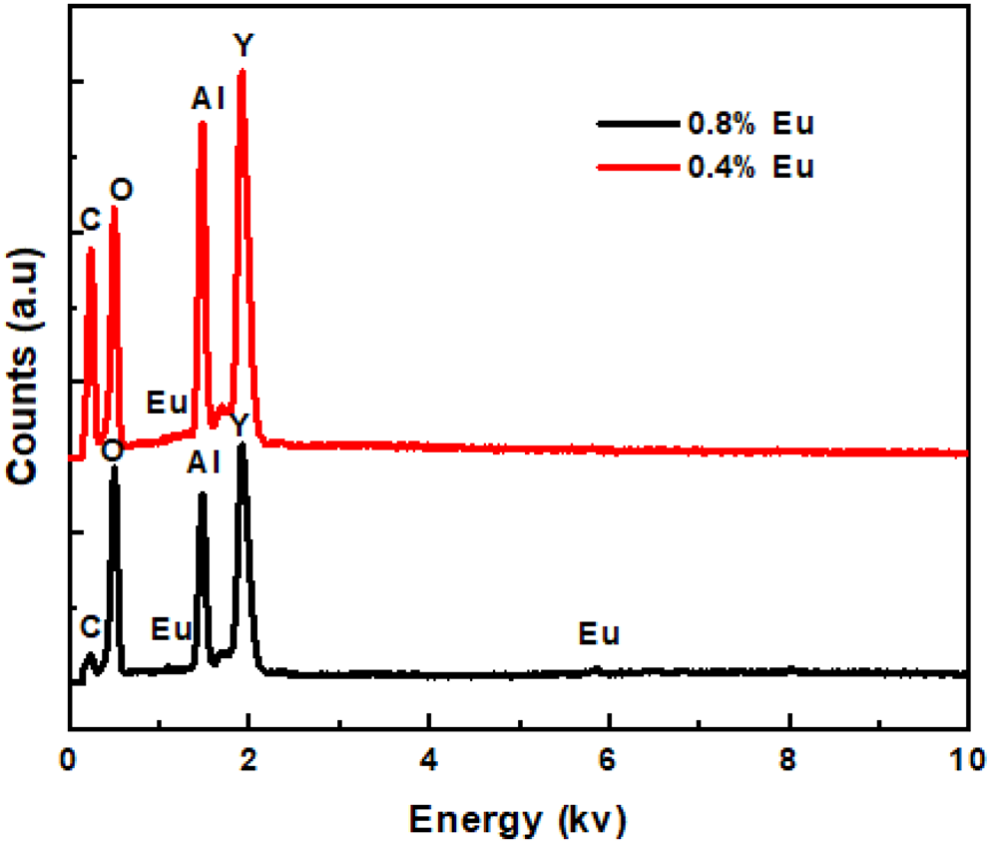
EDS Spectra of Y_3_Al_5_O_12_:Eu^3+^ phosphors

### EDS Analysis

The elemental composition of the Y_3_Al_5_O_12_:Eu^3+^ phosphor is confirmed by the energy dispersive X-ray spectroscopy (EDS) analysis, which is presented in Fig. 5. The EDX spectrum shows peaks corresponding to the expected elements in the Y_3_Al_5_O_12_:Eu^3+^ phosphor. All elements in the Y_3_Al_5_O_12_:Eu^3+^ phosphors are present as shown in Fig. 5. The presence of Y_3_Al_5_O_12_:Eu^3+^ in the sample is confirmed with the Y, Al, O, and Eu peaks. The C peak is coming from the carbon tape on which the sample was mounted.

### Photoluminescence

**Fig. 6:**
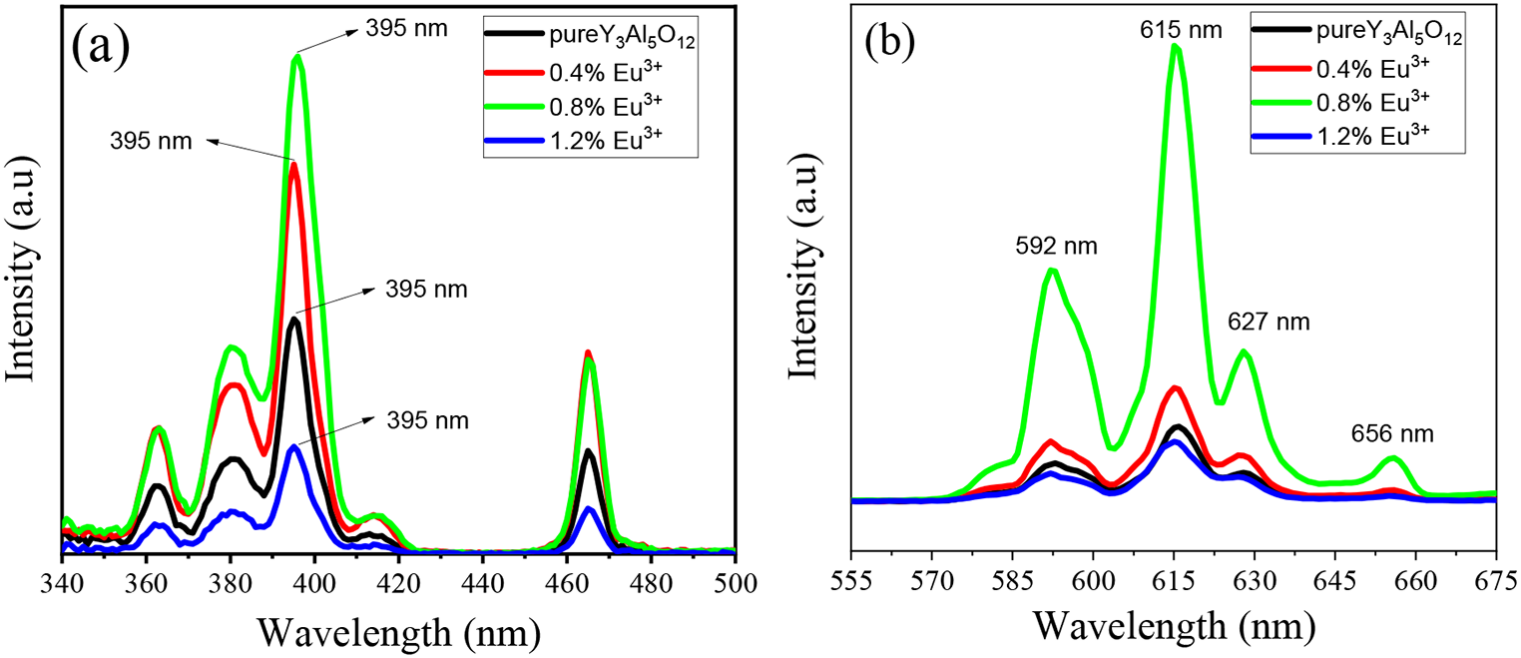
(**a**) Excitation spectra of Y_3_Al_5_O_12_:Eu^3+^ phosphor with different concentration of Eu^3+^ and (**b**) Emission spectra of Y_3_Al_5_O_12_:Eu^3+^ phosphor with different Eu^3+^ concentration

The excitation and emission spectra of the Y_3_Al_5_O_12_:Eu^3+^ phosphors with varying Eu³⁺ concentrations are presented in Fig. 6a and b, respectively. These spectral analyses provide valuable information regarding the luminescent properties and the energy transfer mechanisms within the phosphor material.

#### Excitation Spectra

Figure 6a displays the excitation spectra of Y_3_Al_5_O_12_:Eu^3+^ phosphor, monitored at an emission wavelength of 615 nm. The excitation spectra provide insight into the wavelengths required to excite the phosphor, leading to the observed emission. In the short ultraviolet (UV) region, a broad band is observed, which corresponds to the charge transfer band between the Eu^3+^ ion and the O^2−^ ions in the lattice [24]. This transition arises from the interaction between the Eu^3+^ ions and its surrounding O^2−^ ions. The charge transfer band is crucial for the efficient excitation of Eu^3+^, and it typically lies in the UV region. This band is significant as it provides a pathway for exciting the phosphor through an electron transfer mechanism from O^2−^ to Eu^3+^. In the longer wavelength region of the spectrum, multiple bands are observed, which correspond to the f-f transitions within the Eu^3+^ 4f^6^ electronic configuration [25]. These transitions are characteristic of the Eu³⁺ ion and are responsible for the material’s luminescent properties. Among these, the most intense band appears at 395 nm, which is attributed to the ^7^F_0_-^5^L_6_ transition. This band is the strongest excitation peak in the spectra, confirming that the phosphor is most efficiently excited at this wavelength.

#### Emission Spectra

Figure 6b presents the emission spectra of Y_3_Al_5_O_12_:Eu^3+^ phosphors prepared with different concentrations of Eu^3+^. The emission spectra were recorded after excitation at the most intense peak of 395 nm. The emission spectra primarily fall within the red spectral region, which is characteristic of Eu^3+^ activated phosphors [26, 27]. Three distinct emission peaks are observed at 592 nm, 615 nm, and 627 nm. The peak correspond to the 592 nm is attributed to the ^5^D_0_→^7^F_1_ transition, which is the magnetic dipole transition. The ^5^D_0_→^7^F_1_ transition is typically observed in Eu³⁺ doped materials and results in a red-orange emission [28]. The peak correspond to 615 nm: The ^5^D_0_→^7^F_2_ transition, which is an electric dipole transition, produces the intense red emission at 615 nm [29]. This transition is often the most intense in Eu^3+^ activated phosphors and is responsible for the characteristic red color emission, commonly associated with Eu³⁺ luminescence. For the 627 nm the ^5^D_0_→^7^F_3_ transition results in a slightly longer wavelength emission at 628 nm. This transition is less intense compared to the 615 nm emission but still contributes to the overall red emission observed in the spectrum.

When the Eu^3+^ concentration is raised from 0.4 mol% to 0.8 mol%, a significant increase in the emission intensity is observed. This enhancement is attributed to the higher availability of Eu^3+^ ions, leading to a stronger interaction between the host material and the Eu^3+^ ions [30]. The efficient energy transfer from the host lattice to the Eu^3+^ ions at these concentrations allows for a more pronounced emission. However, as the Eu^3+^ concentration increases further to 1.2 mol%, a decrease in the PL intensity is noted. This decline can be attributed to concentration quenching, a well-known phenomenon that occurs when the concentration of activator ions becomes too high [31, 32]. As the Eu^3+^ concentration increases to 1.2%, the distance between the dopant ions decreases, leading to enhanced non-radiative energy transfer between adjacent ions. This results in a reduction in the overall PL intensity, which is due to a concentration quenching effect. While the structural characterization at 1.2% concentrations shows amorphous nature, the primary cause of the intensity decrease is concentration quenching rather than the amorphousness of the material.

At elevated concentrations of Eu^3+^, non-radiative energy transfer processes become more significant. These processes include dipole-dipole interactions between neighboring Eu^3+^ ions, which result in energy loss through non-radiative channels rather than radiative recombination. The increase in dipole-dipole interactions between adjacent Eu^3+^ ions reduces the efficiency of energy transfer to the luminescent centers, leading to a decrease in emission intensity. These results highlight the critical role of optimizing the Eu^3+^ concentration for achieving high luminescent efficiency in phosphor materials.

**Table 1:** Decay constants of Y_3_Al_5_O_12_:Eu^3+^ phosphor with different Eu concentrations

Eu^3+^	0.0%	0.4%	0.8%	1.2%
Component		Decay constants		
$\:{\tau\:}_{1}$	0.08	0.71	0.84	0.14
$\:{\tau\:}_{2}$	0.93	2.17	2.21	1.54
$\:{\tau\:}_{3}$	2.20	3.10	4.47	2.45

**Fig. 7:**
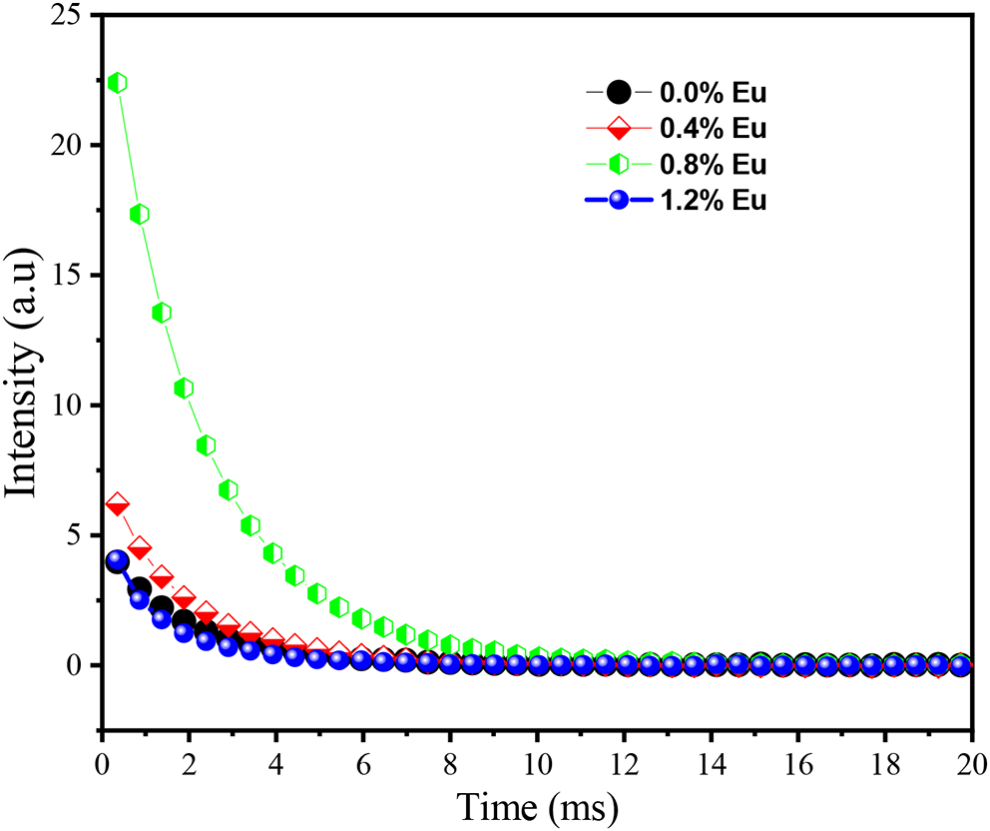
Decay curves of Y_3_Al_5_O_12_:Eu^3+^ phosphors

### Decay Curve

Figure 7 shows the decay curves of Y_3_Al_5_O_12_:Eu^3+^ phosphor with different concentration of Eu. The decay behaviour can be expressed by [33]:

1 $$\:I={A}_{1}{e}^{-\frac{t}{{\tau\:}_{1}}}+{A}_{2}{e}^{-\frac{t}{{\tau\:}_{2}}}+\:\:{A}_{3}{e}^{-\frac{t}{{\tau\:}_{3}}}$$

where, *I* represent the phosphorescent intensity, $\:{A}_{1}$ and $\:{A}_{2}$ are constants, $\:t$ is time, $\:{\tau\:}_{1}$, $\:{\tau\:}_{2}$ and $\:{\tau\:}_{3}$ are the decay times for exponential components. The effect of europium concentration on the photoluminescence decay characteristics of Y_3_Al_5_O_12_:Eu^3+^ phosphors can be understood by analyzing the decay parameters $\:{\tau\:}_{1}$, $\:{\tau\:}_{2}$ and $\:{\tau\:}_{3}$, which are provided in Table 1. The results show that the phosphorescence decay curve initially decreases rapidly and then at a slower rate which is a behavior typical of phosphors that exhibit both fast and slow decay components. At lower concentrations of Eu^3+^ 0.4 mol% and 0.8 mol%, the decay curve shows a significant increase in intensity, indicating enhanced luminescent properties.

As the Eu^3+^ concentration increases within this range, more Eu^3+^ ions are available for energy transfer and radiative emission, leading to a higher overall emission intensity. However, when the concentration of Eu^3+^ reaches higher levels, specifically at 1.2 mol%, the phosphorescence intensity decreases significantly. This is due to concentration quenching effects, which result in a lower overall brightness of the phosphor. At higher concentrations, the Eu^3+^ ions are closer together, leading to increased non-radiative energy transfer between neighboring Eu^3+^ ions, which reduces the efficiency of radiative emission and thus lowers the phosphorescence intensity. Consequently, the phosphor doped with a high concentration of 1.4 mol% Eu^3+^ exhibits a lower intensity and a more rapid decay, indicating poorer brightness performance.

## Conclusions

In this work, red-emitting Y_3_Al_5_O_12_:Eu^3+^ phosphor was successfully synthesized using the solution-combustion method. The X-ray diffraction (XRD) analysis confirmed the formation of a well-crystallized Y_3_Al_5_O_12_ structure. Scanning electron microscopy (SEM) images revealed the agglomerate structure of the synthesized phosphor, while Fourier-transform infrared (FTIR) spectra confirmed the presence of all the key chemical bonds in the Y_3_Al_5_O_12_:Eu^3+^ phosphor. Photoluminescence (PL) spectra demonstrated characteristic emission peaks at 592 nm, 615 nm, and 628 nm, corresponding to transitions from the ^5^D_0_ excited state to the ^7^F_1_, ^7^F_2_, and ^7^F_3_ states of Eu³⁺, with the maximum emission observed near 615 nm. The study showed that the luminescence intensity of the phosphor increased with europium doping concentrations up to 0.8%, beyond which concentration quenching occurred, leading to a decrease in emission intensity at higher doping levels (1.2%). These findings highlight the potential of Y_3_Al_5_O_12_:Eu^3+^ as an efficient red-emitting phosphor and emphasize the importance of optimizing doping concentrations for improved luminescent performance. Finally, we recommend that a further quantum efficiency measurement of the prepared materials to identify its practical applications in luminescence.

## Data Availability

Data sets generated during the current study are available from the corresponding author on reasonable request.
